# Synergistic apoptosis following endoplasmic reticulum stress aggravation in mucinous colon cancer

**DOI:** 10.1186/s13023-020-01499-1

**Published:** 2020-08-18

**Authors:** Ashok K. Dilly, Brendon D. Honick, Yong J. Lee, David L. Bartlett, Haroon A. Choudry

**Affiliations:** 1 Department of Surgery, University of Pittsburgh Medical Center, Hillman Cancer Center, 5150 Centre Avenue, Suite 414, Pittsburgh, PA 15232 USA; 2 Department of Pharmacology & Chemical Biology, University of Pittsburgh Medical Center, Pittsburgh, PA 15232 USA

**Keywords:** MUC2, Mucinous colon cancer, Xenograft, Colonoids, Endoplasmic reticulum stress

## Abstract

**Background:**

Mucinous colon cancers (MCC) are characterized by abundant production of mucin 2 (MUC2) protein and are less sensitive to standard systemic chemotherapy. We postulated that severe/persistent endoplasmic reticulum stress (ERS) aggravation in MCC would overwhelm compensatory cytoprotective pathways and induce apoptosis.

**Results:**

Basal levels of ERS markers were higher in MCC and dnTCF-LS174T cells than non-mucinous tumors and these levels were significantly increased by combinatorial treatment with ERS aggravators celecoxib + orlistat. Combination treatment inhibited cell viability and synergistically induced apoptosis. Treatment-induced cell death was ERS-dependent, apoptotic pathways were not activated following knockdown of ERS protein CHOP. Dual drug treatment significantly reduced mucinous tumor growth in vivo and induced ERS and apoptosis, consistent with in vitro experiments.

**Conclusions:**

Novel therapies are needed since MCC are more resistant to standard systemic chemotherapy. This study suggests ERS aggravation is a viable therapeutic strategy to reduce tumor growth in MCC.

## Background

Mucinous colon cancers (MCC) account for only 10–15% of colorectal cancers in the USA each year (~ 15,000–22,000 cases/year). They arise from rapidly proliferating neoplastic cells with goblet cell-like features that produce large quantities of mucin 2 (MUC2) protein [1–3]. From a treatment standpoint, they are less responsive to systemic chemotherapy in the neoadjuvant and palliative setting. Chemoresistance of MCC has been attributed to the abundant extracellular MUC2 protein that may act as a barrier against drug delivery or immune infiltration, and forms an immunosuppressive/hypoxic microenvironment that impairs treatment efficacy and allows cancer cells to thrive [4]. Therefore, our prior research has focused on targeted therapies that simultaneously inhibit MUC2 production and induce mechanisms for neoplastic cell death [5–9].

The endoplasmic reticulum (ER) is a major site for biosynthesis, post-translational modification and proper folding of proteins like MUC2 that are destined to be secreted from cells [10]. Proteins that fail to undergo correct folding/maturation undergo ER-associated protein degradation (ERAD) via ubiquitin/proteasome- and autophagy-mediated pathways. A variety of conditions that disrupt normal protein processing (e.g. calcium imbalance, hypoxia) can overwhelm the ability of cells to maintain proper protein processing in the ER, thereby triggering ER stress (ERS) and its associated molecular signaling pathways known as the unfolded protein response (UPR). The UPR pathways represent a coordinated effort by cells to decrease overall protein synthesis and improve protein folding/modification or degradation. However, severe and persistent ERS can overwhelm these protective mechanisms and trigger molecular pathways associated with cell death [11–18]. We therefore hypothesized that mucinous colon cancers, characterized by high MUC2 protein turnover, would exhibit elevated basal ERS levels and be vulnerable to therapies that aggravate ERS, thereby inducing ERS-associated cell death pathways.

During low to moderate levels of ERS various pro-survival UPR pathway proteins are activated. These include heat shock protein GRP78 (glucose regulated protein 78, also called BiP, immunoglobulin heavy chain-binding protein) and three ER transmembrane proteins PERK (protein kinase activated by double-stranded RNA-like ER kinase), IRE1 (inositol-requiring enzyme 1) and ATF6 (activating transcription factor 6). During ERS, GRP78 disassociates from these UPR proteins to chaperone terminally misfolded proteins for degradation. At the same time, the freed UPR proteins become activated and initiate signaling pathways that serve to correct or neutralize ERS. Conversely, during severe and persistent ERS pro-apoptotic UPR signaling pathways are activated. These include elevated expression of transcription factor CHOP (C/EBP homologous protein, also called GADD153), inhibition of anti-apoptotic proteins (e.g. Bcl-2), stimulation of pro-apoptotic BH3-only proteins (e.g. BIM) and activation of caspases [10, 17, 18].

In this study, we first characterized basal ERS levels in MCC and then investigated whether aggravation of ERS in these tumors would induce ERS-associated cell death. We studied a combination of two federal drug administration (FDA) approved drugs to aggravate ERS, celecoxib (ERS aggravation by inhibition of non-cyclooxygenase [COX-2] target sarcoplasmic/endoplasmic reticulum calcium ATPase [SERCA]) and orlistat (ERS activation by fatty acid synthase [FASN] inhibition) [15, 19–24]. SERCA inhibition on the ER membrane activates ERS by disrupting calcium homeostasis within the ER, while FASN inhibition triggers ERS by a variety of mechanisms including the disruption of protein lipidation, an important process for normal protein folding, stability, membrane association, localization, trafficking and secretion [25–32]. Additional rationale for the use of these drugs include the basal over-expression of COX-2 and FASN in MCC and prior studies demonstrating inhibition of MUC2 expression/secretion by targeting these two cancer pathways [7, 33, 34]. Both celecoxib and FASN inhibitors have demonstrated effective in vitro and in vivo cellular growth suppression in a variety of malignancies including colorectal cancer, however clinical trials of these drugs have been disappointing [19, 25, 29]. We postulate that the irrational use of such drugs against unselected cancers in clinical trials is likely responsible for lack of clinical efficacy to date and that MCC are more likely to be susceptible to such targeted therapies given their high basal ERS levels.

## Results

### MCC and high MUC2 producing cells exhibit elevated basal ERS that correlates with MUC2 expression levels

**Fig. 1:**
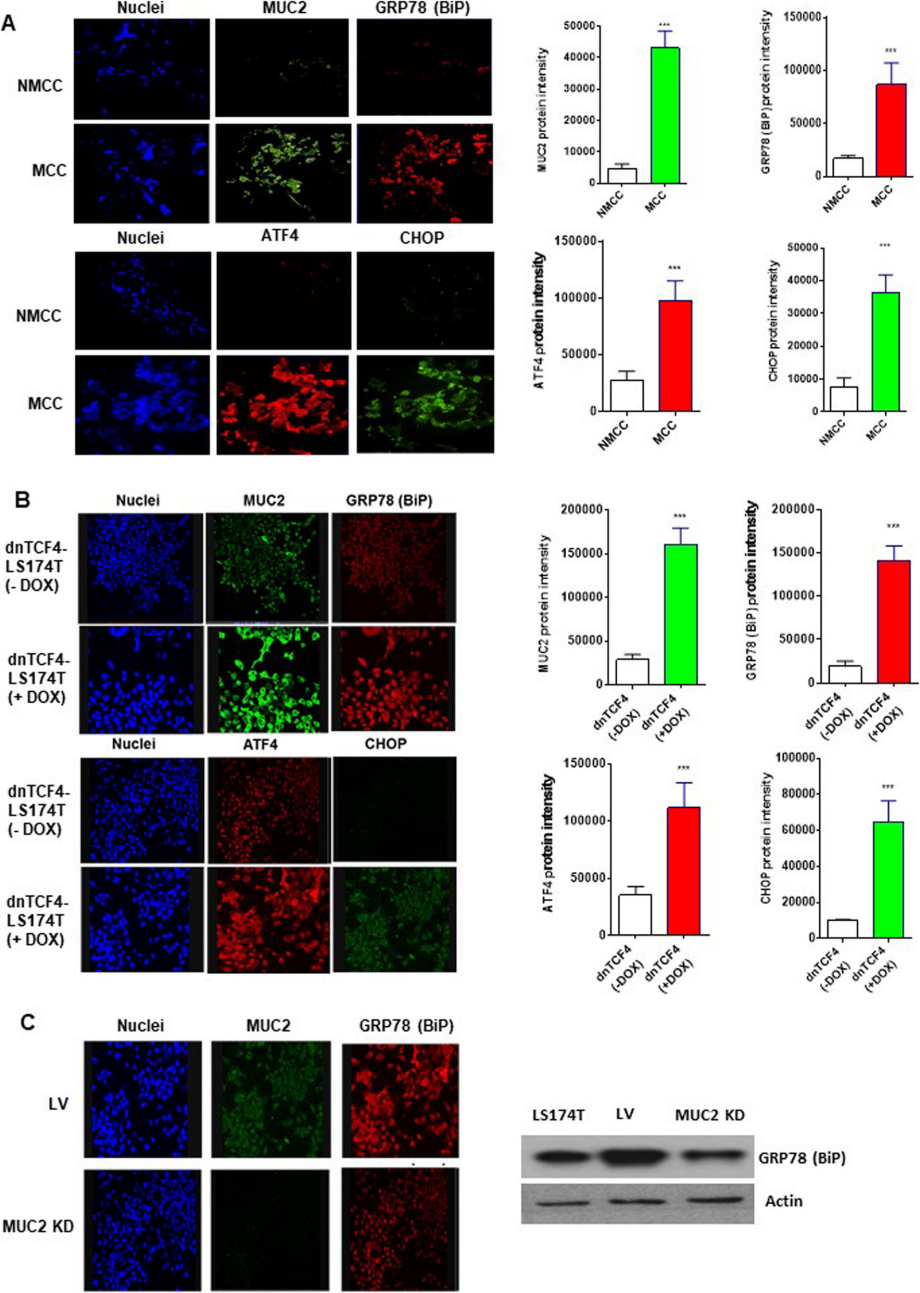
Mucinous colon cancers and high MUC2 producing cell lines exhibit elevated basal ERS that correlates with MUC2 expression levels. **a** Representative pictures from immunofluorescence (IF) analysis of human explant tissue comparing MUC2 and ERS proteins (GRP78 [BiP], ATF4, CHOP) between 5 mucinous colon cancer (MCC) and 5 non-MCC (NMCC); the bar graphs demonstrate mean intensity difference. **b** Representative pictures from IF analysis of MUC2 and ERS proteins (GRP78 [BiP], ATF4, CHOP) comparing high-MUC2 expressing cells (dnTCF4-LS174T cells exposed to doxycycline for 48 h) versus low-MUC2 expressing cells (dnTCF4-LS174T cells without doxycycline exposure); the bar graphs demonstrate mean intensity difference. **c** Representative pictures from IF analysis of MUC2 and GRP78 (BiP) comparing stable MUC2 knockdown (KD) in LS174T cells compared to lentiviral (LV) control cells; western blot shows MUC2 protein expression in MUC2 KD cells compared to lentiviral control cells. Error bars represents standard deviation (S.D.) from triplicate experiments (*** *p* < 0.001)

We compared basal ERS levels in explant tissue from patients with MCC and NMCC. We found significantly higher basal MUC2, GRP78 (BiP), ATF4 and CHOP protein expression levels in explant tissue from MCC compared to NMCC (Fig. 1a). Similar results were found when comparing high versus low MUC2 expressing LS174T cells. dnTCF4-LS174T cells exposed to doxycycline for 48 h (high MUC2 expressing cells) expressed higher levels of MUC2 and UPR proteins GRP78 (BiP), ATF4, and CHOP compared to dnTCF4-LS174T cells lacking doxycycline exposure (low MUC2 expressing cells) (Fig. 1b) or wild-type LS174T cells (low MUC2 expressing cells; data not shown).

We confirmed the association of MUC2 expression and UPR pathway activity by demonstrating a decrease in GRP78 (BiP) levels following stable MUC2 knockdown (MUC2 KD) in LS174T cells compared to lentiviral (LV) control cells (Fig. 1c). These data demonstrate higher basal ERS levels in MCC/high MUC2 producing cells compared to NMCC/low mucin producing cells and suggest a correlation between MUC2 expression and cellular basal ERS levels. These results support our hypothesis that MCC, with high basal ERS, may be susceptible to ERS-aggravation as a therapeutic strategy to induce ERS-mediated apoptosis.

### ERS is aggravated by dual celecoxib plus orlistat drug therapy in MCC and correlates with MUC2 expression levels

**Fig. 2:**
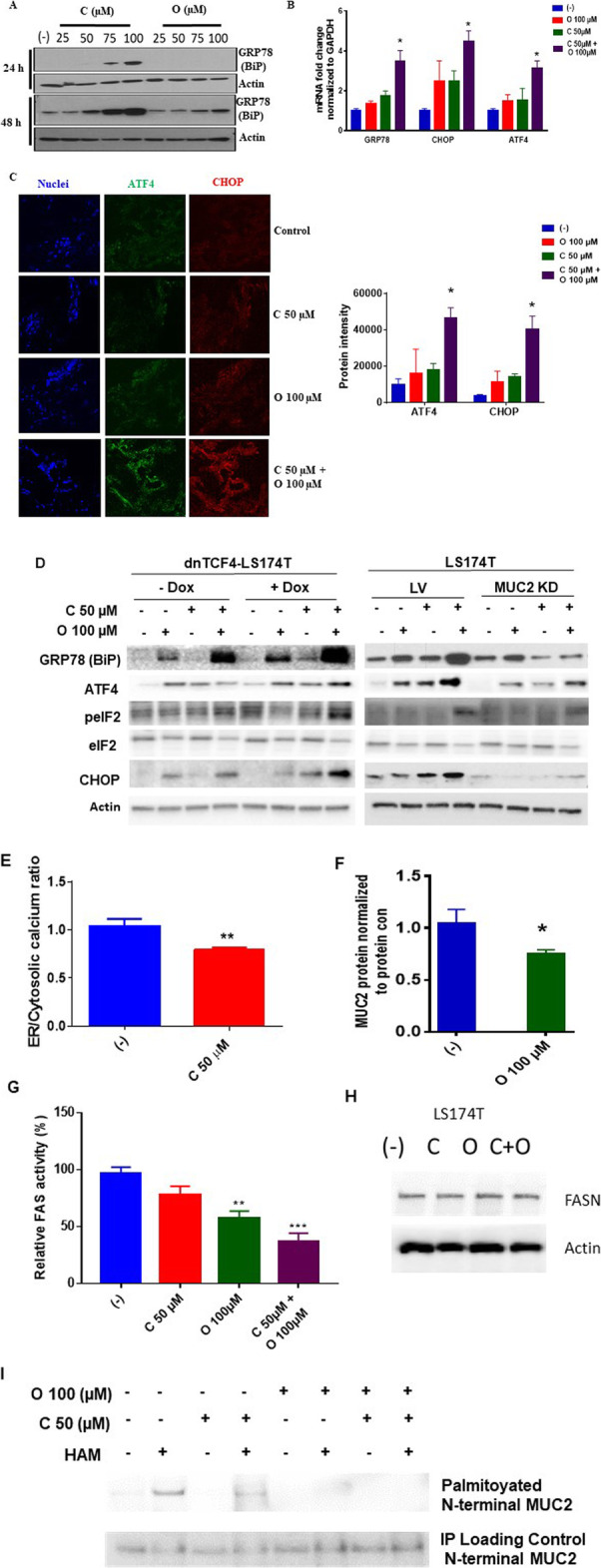
ERS is aggravated by dual celecoxib plus orlistat drug therapy in mucinous colon cancer and correlates with MUC2 expression levels. **a** Western blot assay of ERS protein GRP78 (BiP) following treatment of LS174T cells with celecoxib (0–100 μM) or Orlistat (0–100 μM) for 24 and 48 h. qPCR assay of mRNA expression for ERS markers following treatment with celecoxib, orlistat, or combination for 24 h in LS174T cells **b** and MCC explant tissue **c**; representative pictures of IF assay with bar graph demonstrating mean intensity difference. **d** Western blot assay of ERS markers comparing high MUC2 expressing cells (dnTCF4-LS174T cells exposed to doxycycline for 48 h) versus low MUC2 expressing cells (dnTCF4-LS174T cells without doxycycline exposure) and comparing stable MUC2 KD in LS174T cells compared to LV control cells following treatment with celecoxib, orlistat, or combination for 24 h. **e** Ratio of ER:cytoplasmic calcium concentration at 30 s in LS174T cells following combination treatment (celecoxib + orlistat). **f** ELISA assay of MUC2 secretion from COS-7 cells transfected with pSNMUC2-MG vector expressing MUC2 N-terminal following treatment with celecoxib, orlistat, or combination for 24 h. **g** FASN enzymatic activity assay in LS174T cells following combination treatment (celecoxib + orlistat) for 24 h; and **h** western blot analysis of FASN expression levels. **i** N-terminal MUC2-palmitoylation in COS-7 cells stably expressing MUC2 N-terminal following single or dual drug therapy for 24 h was determined by ABE assay and quantified by western blot assay; hydroxylamine (HAM), a strong reducing agent that cleaves palmitate from cysteine residues, is necessary for biotinylation, the omission of HAM cleavage (HAM -) serves as negative control. Error bars represents standard deviation (S.D.) from triplicate experiments (* *p* < 0.05, ** *p* < 0.01, *** *p* < 0.001)

We demonstrated a dose-dependent increase in UPR protein GRP78 (BiP) levels in LS174T cells when exposed to increasing doses of celecoxib (0–100 μM) or orlistat (0–100 μM) for 24–48 h, consistent with their known role as ERS aggravators (Fig. 2a). Similarly, mRNA expression levels for GRP78 (BiP), CHOP, and ATF4 increased in LS174T cells exposed to combination of celecoxib (50 μM) and orlistat (100 μM) for 24 h (Figs. 2b). Explant tissues derived from MCC demonstrated similar increase in UPR protein levels (ATF4 and CHOP) following combination therapy for 24 h (Fig. 2c).

We demonstrated a significant increase in UPR proteins (GRP78 (BiP), ATF4, peIF2 [phosphorylated-eukaryotic initiation factor 2], and CHOP) in dnTCF4-LS174T cells exposed to doxycycline for 48 h (high MUC2 expressing cells) following treatment with combination of orlistat (100 μM) and celecoxib (50 μM) compared to low mucin producing dnTCF4-LS174T cells lacking doxycycline exposure. Conversely, combination therapy-induced ERS aggravation was significantly reduced following stable MUC2 KD in LS174T cells compared to LV control cells. We found much higher levels of GRP78, ATF4, peIF2, and CHOP in LV control cells exposed to dual drug therapy compared to levels seen in MUC2 KD cells (Figs. 2d). These results suggest that the degree of drug-induced ERS aggravation correlates with MUC2 expression levels and that high mucin producing cells are more susceptible to drug-mediated ERS aggravation.

Treatment of LS174T cells with celecoxib (50 μM) led to rapid decrease in ER/cytoplasmic calcium ratio at 30 s, consistent with inhibition of calcium ATPase channel on the ER membrane, and the likely mechanism for ERS aggravation **(**Fig. 2e). We evaluated changes in MUC2 protein secretion following orlistat treatment, since FASN is essential for the post-translational lipidation and secretion of MUC2 protein. We found that orlistat (100 μM) inhibited MUC2 secretion from COS-7 cells transfected with pSNMUC2-MG vector expressing MUC2 N-terminal (Fig. 2f). Mechanistically, we found that exposure to orlistat reduced FASN enzyme activity (but not enzyme expression levels) (Fig. 2g, h). Moreover, we performed ABE assay in COS-7 cells stably expressing MUC2 N-terminal. Our data demonstrated a significant reduction in MUC2 N-terminal palmitoylation and secretion following orlistat therapy, a likely mechanism for ERS aggravation (Fig. 2i).

### ERS aggravation by celecoxib plus orlistat combination therapy induces ERS-mediated apoptosis

**Fig. 3:**
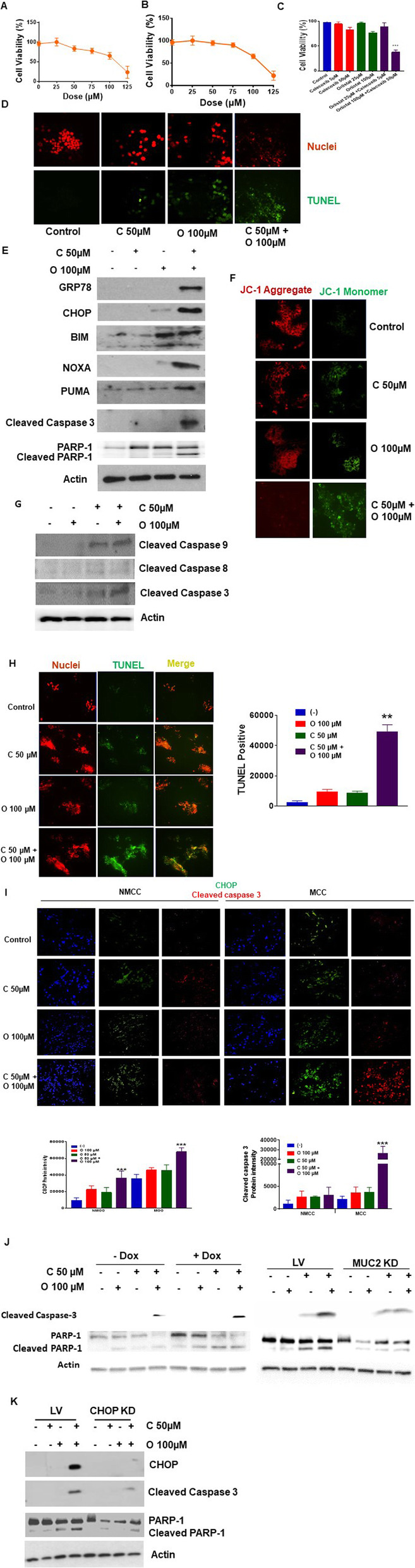
ERS aggravation by celecoxib plus orlistat combination therapy induces ERS-mediated apoptosis. MTS cell proliferation assay in LS174T cells treated with increasing doses of celecoxib (0–125 μM) **a** or orlistat (0–125 μM) **b** or combination of celecoxib and orlistat for 24 h **c**. Representative pictures of TUNEL assay in LS174T cells treated with single or dual drug therapy for 24 h **d**. Western blot analysis for ERS and apoptotic markers at 24 h **e**. **f** LS174T cells were treated with single or dual drug therapy and stained with JC-1. Diffuse green JC1-monomers indicate mitochondrial depolarization (damage), and punctate red JC1-aggregates indicates intact mitochondrial membrane potential (ΔΨm). **g** Western blot assay for caspases in LS174T cells following single and dual drug therapy for 24 h. **h** Representative pictures of TUNEL assay in colonoid cultures from MCC following single and dual drug therapy for 24 h; the bar graph demonstrates mean intensity difference. **i** Representative pictures from immunofluorescence (IF) analysis of human explant tissue comparing caspase 3 and CHOP between 5 MCC and 5 NMCC following single and dual drug therapy for 24 h; the bar graph demonstrates mean intensity difference. **j** Western blot assay of apoptotic markers comparing high-MUC2 expressing cells (dnTCF4-LS174T cells exposed to doxycycline for 48 h) versus low-MUC2 expressing cells (dnTCF4-LS174T cells without doxycycline exposure) and comparing stable MUC2 knockdown (KD) in LS174T cells compared to LV control cells following treatment with celecoxib, orlistat, or combination for 24 h. **k** Western blot assay of CHOP and apoptotic markers comparing stable CHOP knockdown (KD) in LS174T cells compared to LV control cells following treatment with celecoxib, orlistat, or combination for 24 h. Asterisk represents a statistically significant difference compared with the control group (** *p* < 0.01, *** *p* < 0.001)

We treated LS174T cells with celecoxib (0–125 μM) and orlistat (0–125 μM), alone and in combination, at varying doses for 24 h (Fig. 3a-c). The IC50 doses for celecoxib alone and orlistat alone were 108 μM and 109.5 μM, respectively. Cell viability was significantly reduced at 24 h following combination therapy with celecoxib (50 μM) and orlistat (100 μM), demonstrating a combination index of 0.487 (calculated using the computer software Compusyn) suggested synergy between the two drugs in reducing cell viability. Combination therapy induced significant apoptosis, as demonstrated by TUNEL assay at 24 h. (Figs. 3d) LS174T cells exposed to combination therapy with celecoxib (50 μM) and orlistat (100 μM) for 24 h demonstrated significant increase in UPR proteins GRP78 (BiP) and CHOP, pro-apoptotic molecules BIM, NOXA and PUMA and activated (cleaved) caspase 3/PARP-1, while single drug therapy at these doses had minimal effect (Figs. 3e). We assessed changes in mitochondrial transmembrane potential (ΔΨm) following dual drug therapy using mitochondrial membrane-permeant fluorescence dye JC-1. JC-1 aggregates (reduced red-fluorescent J-aggregates) were significantly reduced following co-treatment, consistent with mitochondrial membrane damage and activation of intrinsic apoptosis (Fig. 3f). Involvement of intrinsic mitochondrial apoptotic pathway was confirmed by cleavage of caspase 9/3 but not caspase 8 following dual drug therapy (Fig. 3g). We also confirmed the induction of UPR and apoptotic proteins in colonoid cultures and explant tissue. Exposure to combination of celecoxib (50 μM) and orlistat (100 μM) for 24 h resulted in apoptosis (TUNEL assay) in colonoid cultures (Fig. 3h) and much higher CHOP and cleaved caspase 3 levels in explant tissue from MCC compared to NMCC, suggesting their susceptibility to ERS aggravation and ERS-mediated apoptosis (Fig. 3i).

Similarly, we demonstrated a significant increase in apoptotic markers (PUMA, cleaved caspase 3/PARP-1) in dnTCF4-LS174T cells exposure to doxycycline for 48 h (high MUC2 expressing cells) following combination treatment with orlistat (100 μM) and celecoxib (50 μM) compared to low mucin producing dnTCF4-LS174T cells lacking doxycycline differentiation. Conversely, combination therapy-induced apoptosis was significantly dampened following stable MUC2 KD in LS174T cells compared to LV control cells. We found high levels of PUMA, cleaved caspase 3/PARP-1 in LV control cells exposed to combination of orlistat and celecoxib, while these were suppressed in MUC2 KD cells (Figs. 3j). Our results showed that combination treatment induced apoptosis correlated with MUC2 expression.

To determine whether combination therapy induced apoptosis was ERS dependent, we tested this drug combination in CHOP knockdown LS174T cells (CHOP KD). We demonstrated a significant reduction in cleaved caspase 3/PARP-1 in CHOP KD cells compared to LV control cells (Fig. 3k).

### Combination of celecoxib and orlistat reduced mucinous tumor growth in vivo

**Fig. 4:**
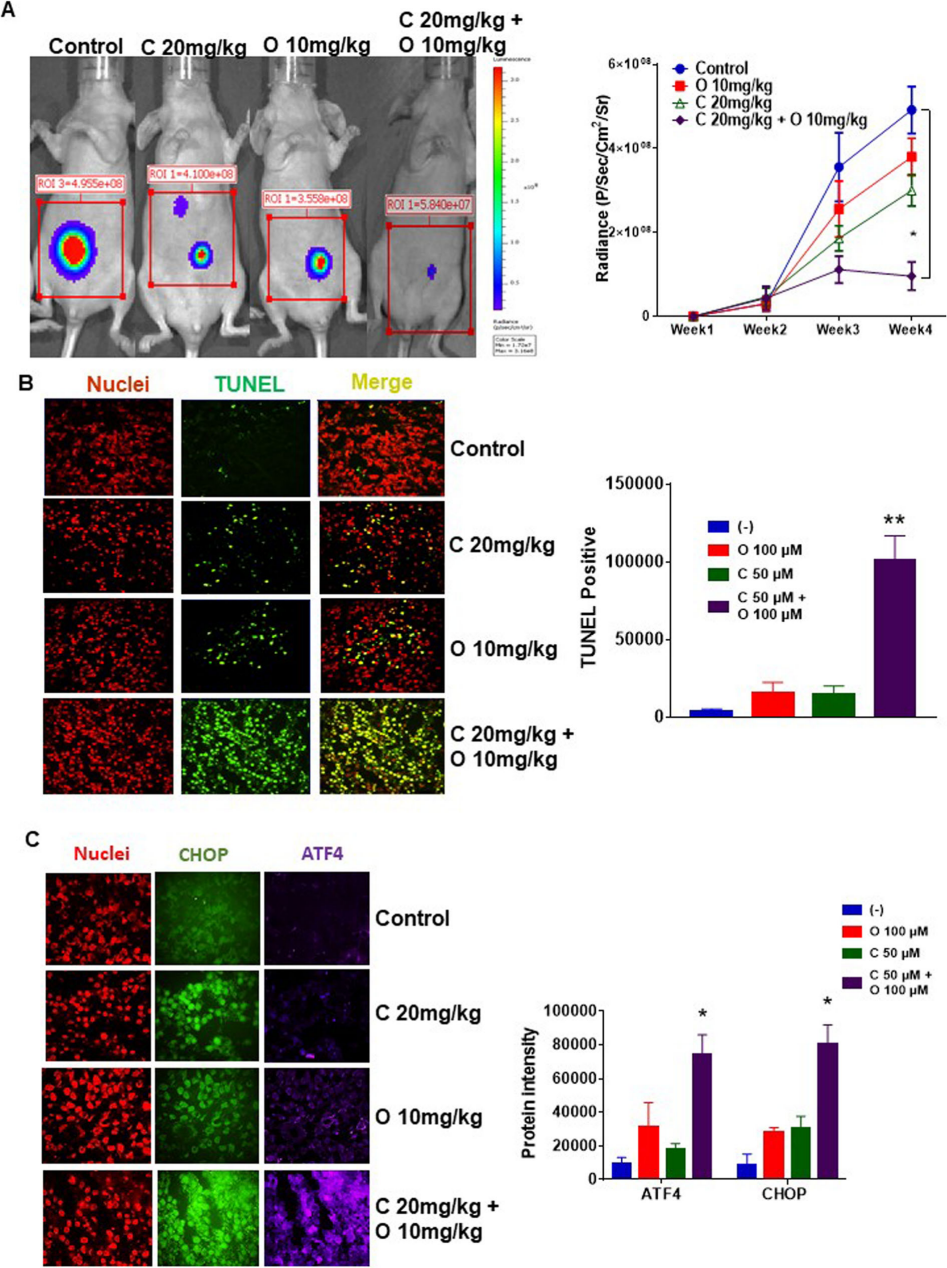
Combination of celecoxib and orlistat reduced mucinous tumor growth in vivo*.* Murine xenograft models of intraperitoneal (IP) luciferase-labelled LS174T cells were treated with PBS alone (control), or celecoxib (C) alone (200 mg/kg b.w.), or orlistat (O) alone (10 mg/kg b.w.), or combination of C + O every other day (starting on day 7 following tumor implantation) until they were euthanized at day 28. Gross intra-abdominal tumor burden is depicted pictorially on day 28**;** serial weekly changes in mean luciferase intensity during treatment for 3 weeks are shown **a**; error bars represent standard error of the mean among the 6 xenografts in each treatment group. Harvested tumor tissue from euthanized mice (day 28) was subjected to TUNEL IF assay **b** and IF for ERS markers **c**; the bar graphs demonstrate mean intensity difference. Asterisk represents a statistically significant difference compared with the control group (* *p* < 0.05, ** *p* < 0.01)

We evaluated the therapeutic efficacy of this combination in vivo using IP PDX models of luciferase-labelled LS174T cells. Seven days following IP tumor inoculation, animals were treated IP with vehicle (PBS), celecoxib (C) alone (20 mg/kg), orlistat (O) alone (10 mg/kg) or combination of celecoxib and orlistat (C + O), every other day for 3 weeks (6 animals per group). Drug dose selection for IP celecoxib and orlistat was based on prior publications and our pilot studies [7, 35]. Treatment-related drug toxicity was not encountered in the in vivo experiments. Dual drug therapy resulted in significant reduction in mucinous tumor growth, compared to either drug alone, as demonstrated by luciferase intensity measured by IVIS bioluminescent Imaging System (Fig. 4a). Tumor tissue harvested from euthanized animals following 3 weeks of therapy demonstrated a significant increase in apoptosis (TUNEL positive staining) with dual therapy (Fig. 4b). Changes in UPR protein levels (CHOP and ATF) were consistent with those seen in in vitro studies (Fig. 4c).

## Discussion

MCC are a unique histologic subtype in which greater than 50% of the tumor mass is composed of extracellular MUC2 protein [3]. They demonstrate unique clinical phenotype and molecular genotype when compared to their non-mucinous counterparts (NMCC). Clinically, MCC are more likely to occur in younger patients, have a predilection for the proximal colon, are bulkier at presentation, and have a higher propensity for peritoneal and distant lymph node metastases. Molecularly, they tend to follow the serrated pathway for carcinogenesis, and are more likely to have RAS/GNAS mutations, microsatellite instability (MSI) and cpG island methylator phenotype (CIMP positive) [1, 36].

In general, many molecular-targeted therapies have demonstrated disappointing results against advanced solid cancers in clinical trials, despite promising preclinical data. This may largely be attributed to irrational use of targeted drugs against unselected cancers. Major considerations to improve the efficacy of targeted therapies include identification of ideal drugs and drug combinations, optimizing dosing schedules, and enriching for patient factors (e.g., clinical and molecular phenotypes and genotypes) that are more likely to respond to specific targeted therapies. In this study, we focused on specific vulnerabilities of mucinous tumors that may make them susceptible to specific targeted therapies, thereby applying a rational approach to treating well-selected cancers. We hypothesized that high basal ERS of MCC would make them vulnerable to ERS aggravation and ERS-mediated cell death processes. In this study we aggravated ERS via two well-established pathways, SERCA inhibition (celecoxib) to impair normal calcium homeostasis and FASN inhibition (orlistat) to exacerbate protein misfolding by inhibiting normal MUC2 secretion. Our rationale for utilizing celecoxib was that SERCA is a known non-COX-2 target of celecoxib and would therefore aggravate ERS, while at the same time celecoxib has been shown to inhibit MUC2 expression and decrease mucinous tumor growth in ex vivo and in vivo models of mucinous colon/appendix cancers [10, 13, 15]. Our rationale for utilizing orlistat was that it inhibits FASN (required for MUC2 secretion) which would induce ERS, while at the same time reducing mucinous tumor growth [25–27, 32]. Moreover, there is considerable crosstalk between COX-2 and FASN signaling pathways; FASN plays a role in the conversion of excess carbohydrates to arachidonic acid (a polyunsaturated fatty acid), COX-2 is responsible for converting arachidonic acid to prostanoids, and non-COX-2 target PDK-1 regulates FASN activity [33, 34]. Both COX-2 and FASN activities modulate cancer signaling and have been shown to modulate cell proliferation and survival [25, 29, 37].

In this study, we first demonstrated that MCC and high MUC2 producing cells (dnTCF4-LS174T exposed to doxycycline) had elevated basal ERS levels compared to NMCC and low MUC2 producing cancer cells, and that the mucinous nature of these tumors (excessive MUC2 protein production) was responsible for the high basal ERS levels. This laid the foundation for our targeted therapeutic approach since severe and persistent ERS (ERS aggravation) can overwhelm normal protective mechanisms (UPR) to cope with this excessive stress and trigger molecular pathways associated with cell death. We then tested the therapeutic efficacy of dual drug therapy with celecoxib and orlistat since they are both ERS aggravators, have demonstrated preclinical efficacy against a variety of cancers, have mechanisms of action that would inhibit MUC2 protein expression/secretion, and target COX-2/FASN cancer pathways with significant crosstalk. ERS aggravation as a potential mechanism to induce cell death in cancers has been tested in cancer models by other investigators and we felt this approach would be especially effective in mucinous tumors given the elevated basal ERS levels (i.e. rational application in well-selected tumors). Celecoxib is an FDA approved drug used to prevent cancer progression in high-risk patients with familial adenomatous polyposis (FAP) and continues to be investigated in clinical trials to improve the efficacy of standard chemotherapeutic drugs [19, 37]. Orlistat, a reduced form of the natural product lipstatin, is FDA approved as an oral anti-obesity drug since it inhibits gastric/pancreatic lipases and thereby decreases absorption. Due to its poor oral bioavailability, intravenous formulations of orlistat are under development and have shown efficacy in preclinical models, while other FASN inhibitors continue to be tested as anti-cancer drugs in clinical trials [29]. In our study, combination treatment (celecoxib + orlistat) aggravated ERS and synergistically induced markers associated with apoptotic cell death in MCC explants tissues and MCC-derived colonoid cultures. Treatment-induced cell death was ERS-dependent since pro-apoptotic cell death pathways were not activated following knockdown of ERS protein CHOP. Dual drug treatment significantly reduced mucinous tumor growth in vivo and induced markers of ERS and apoptosis, consistent with in vitro experiments.

Our study has a number of limitations. Mechanistic studies assessing changes in ubiquitin-proteosome and autophagy markers following ERS aggravation would be important to elucidate cellular changes that underlie transition from cytoprotective UPR to cytodestructive apoptotic pathways. Such studies would also help quantitate the magnitude of ERS aggravation required to induce this transition. Additional studies with other ERS aggravating agents would confirm findings of this study and the proposed therapeutic approach for MCC. For the in vivo studies, celecoxib and orlistat were administered IP which is not ideal for clinical trials. While celecoxib may be given orally, orlistat has poor bioavailability. Intravenous formulations of orlistat have been developed and other parenterally administered FASN inhibitors are in clinical trials [29]. These alternate drugs and routes of administration will need to be tested in our models.

## Conclusions

In summary, we found that combination of celecoxib and orlistat significantly aggravated ERS and this was more pronounced in MCC and high MUC2 producing cells (dnTCF4-LS174T exposed to doxycycline) compared to NMCC and low MUC2 producing cancers, and that the mucinous nature of these tumors (excessive MUC2 protein production) was responsible for the high aggravated ERS levels. We also demonstrated that ERS aggravation by dual therapy with celecoxib and orlistat induced apoptosis and suppressed mucinous tumor growth, providing a preclinical rationale for the use of this therapeutic strategy in the clinical setting.

## Materials and methods

### Reagents

DMEM (Dulbecco’s Modified Eagle’s Medium) was obtained from Invitrogen (Carlsbad, CA). Fetal bovine serum (FBS) was obtained from Hyclone Laboratories (Logan, UT). Cell-culture plates were purchased from Denville Scientific (Charlotte, NC). Celecoxib and Orlistat were obtained from Cayman Chemical (Ann Arbor, MI). Cell Titer 96 Aqueous One Solution Cell Proliferation assay was obtained from Promega (Madison, WI). The enhanced chemiluminescence reagents (ECL) kit and Pierce BCA protein assay were obtained from ThermoScientific (Rockford, IL). BD Pharmigen FITC Annexin V Apoptosis Detection Kit I was obtained from BD Biosciences (San Jose, CA). Control, MUC2, and CHOP shRNA lentiviral particles were obtained from Santa Cruz Biotechnology (Santa Cruz, CA). For western blotting Beta-Actin (A1978) was obtained from Sigma Aldrich, St. Louis, MO; PARP-1 (CST 46D11), GRP78 (CST C50B12), ATF4 (CST D4B8), PUMA (CST D30C10), NOXA (CST D8L7U), CHOP (CSTL63F7), and Cleaved Caspase 3(CST Asp175) were obtained from Cell Signaling Technologies (Danvers, MA). SYTOX Orange for nucleic acid labeling was obtained from Life Technologies (Grand Island, NY). Anti-rabbit and anti-mouse horseradish peroxidase (HRP)-conjugated secondary antibodies were purchased from Jackson Immunology Research (West Grove, PA).

### Cell culture and treatment

LS174T cell line (MUC2 producing colon cancer cells with goblet cell-like characteristics) was obtained from American Type Culture Collection. High MUC2 producing cells (dnTCF4 cell line under Tet-on control system) was kindly gifted by Hans Clevers (Utrecht, Netherlands) are able to differentiate into goblet-like cells following doxycycline induction 48–96 h [38]. Cells were grown in DMEM (supplemented with 10% fetal bovine serum, 100I/U penicillin and 100 μg/ml streptomycin) in 5% CO_2_ at 37 °C in culture plates.

### Stable cell line generation

LS174T cells were incubated with MUC2 or CHOP short-hairpin RNA (shRNA, h) lentiviral particles and 5 μg/mL final concentration polybrene (Sigma-Aldrich, #9268). Following 24 h of incubation, the medium was replaced with complete DMEM for 24 h and then puromycin was added at a final concentration of 3 μg/mL. Cell were sub-cultured for 3 weeks under puromycin selection to eliminate non-transduced cells.

COS-7 cells were transfected with pSNMUC2-MG vector expressing MUC2 N-terminal (a gift from Gunnar. Hansson, Gothenburg, Sweden) [39]. Following overnight incubation, the medium was replaced with complete DMEM for 24 h and then G418 was added at a final concentration of 500 microgram per ml to eliminate non-transfected cells.

### Confocal imaging

Tumor tissues were embedded in OCT medium-containing cryomolds and immediately frozen in 2-methyl-butane. Then, 5 μm frozen tissue sections were cut using a cryostat and layered on super frost plus slides. Cells were grown on glass coverslips in 12 well plates for in vitro experiments. The covers were incubated in 4% paraformaldehyde for 15 min and then washed and blocked for 60 min at room temperature. The cells were then incubated with MUC2 or GRP78 or CHOP antibody. They were then washed 5 times with 1X PBS and incubated with anti-rabbit Alexa 647 and anti-mouse Alexa 488 and SYTOX orange for nucleic acid staining at room temperature for 30 min. Repeat 5 times washing with 1X PBS was performed. Glass slips were mounted on slides using ProLiong Gold antifade solution from Invitrogen (Life Technologies, Grand Island, NY). Confocal images were taken from 10 different fields at random at X63 magnification using a LEICA confocal TCS SL DMRE microscope.

### Cell proliferation assays

Cell lines were counted and plated in a 96-well plate overnight. The next day the cells were treated with varying concentrations of Celecoxib and Orlistat for 24 or 48 h. Following this treatment cell viability was determined by CellTiter 96 Aqueous One Solution Cell Proliferation (MTS) Assay according to the manufacturer’s protocol (Promega, Madison, WI). Cells were incubated with the combined solution of a tetrazolium compound MTS [3-(4,5-dimethylthiazol-2-yl)-5-(3-carboxymethoxyphenyl)-2-(4-sulfophenyl)-2H-trazolium, inner salt] and electron coupling reagent PMS (phenazine methosulfate) for 2 h at 37 °C. The absorbance of the product was measured at 490 mm directly with an enzyme-linked immunosorbent assay plate reader. All cell treatments were performed in triplicate.

### Apoptosis analysis

Cells were counted and plated into a 24 well plate for 48–72 h. Cells were then treated with varying concentrations of Celecoxib and Orlistat alone and in combination for 24 to 48 h. Following treatment cells and medium were collected in 5 ml culture tubes and apoptosis was analyzed using flow cytometry and BD Pharmigen FITC Annexin V Apoptosis Detection Kit I (BD Biosciences, San Jose, CA) per manufacturer’s protocol. Briefly, cells were washed twice with cold PBS and then resuspended in 100 μl of 1X Binding Buffer. Five μl of both FITC Annexin V and propidium iodide (PI) were added two each tube. For each cell line one tube was collected and resuspended in 100 μl 1X binding buffer and incubated unstained; without Annexin V and PI; or with only Annexin V or PI. Cells were incubated for 15 min at room temperature in the dark. Following this the samples were analyzed by flow cytometry using an Accuri C6 Flow Cytometer. Combination index was measured using the CompuSyn software (Combo Syn, Inc. Paramus, NJ).

### Western blotting

Cells were treated for 24 h with varying concentrations of Celecoxib and Orlistat alone or in combination. Following treatment cells were scrapped and collected in 15 ml conical tubes and kept on ice. A cell pellet was collected by centrifugation and washed twice with cold PBS. Cell pellets were resuspended in 1x RIPA solution (Cell Signaling Technology) with 1x Protease inhibitor (cOmplete Mini, Sigma Aldrich) in PBS and lysis was performed using sonication and then centrifuged for 10 min at 14000×g at 4 °C. Supernatant was collected. Protein concentration was determined in non-reduced samples using BCA reagent (Thermo Scientific). Protein was run on 4–20% SDS gels (Mini-PROTEAN TGX Gels, Bio Rad) and transferred to PVDF membranes. Blocking was performed with 5% milk in TBST. Membranes were incubated with the primary antibodies: PARP-1 (CST 46D11), Actin (Sigma A1978, St. Louis, MO), GRP78 (CST C50B12), ATF4 (CST D4B8), PUMA (CST D30C10), NOXA (CST D8L7U), CHOP (CST L63F7), and Cleaved Caspase 3(CST Asp175). Membranes were incubated with the appropriate Rabbit or Mouse secondary antibody (Jackson Immunology Research, West Grove, PA). Protein was detected SuperSignal West Pico PLUS Chemiluminescent Substrate (ThermoFischer) per manufacturer’s protocol using an equal mix of enhanced chemiluminescent horseradish peroxidase and Super Signal West Pico PLUS substrate. Blots were then developed using x-ray film.

### Real-time PCR

Total RNA was isolated from LS174T cells using Qiagen RNA isolation kit. The cDNA was prepared using the Quanta cDNA synthesis kit. Real-time PCR was then carried out using an ABI Prism SDS 7000 Cycler system, using commercially available primers and probe obtained from ABI for specific cDNA, for 40 cycles at 95 °C for 15 s. All PCR reactions were performed in triplicate, the house keeping gene glyceraldehyde 3-phosphate dehydrogenase (GAPDH) was used as a reference gene for the mRNA levels of genes of interest.

### Terminal deoxynucleotidyl transferase dUTP nick end labeling (TUNEL) assay

In situ BrdU-Red DNA Fragmentation kit was used to detect apoptosis in frozen tissues. Br-dUTP staining was used to detect the DNA strand breaks. Briefly, tumor crypts sections were deparaffinized, permeabilized using proteinase K, DNA strand breaks were end-labeled with terminal transferase, and then visualized using fluorescence microscopy.

### Colonic crypt isolation and culture

Fresh primary mucinous colon cancer (MCC) and non-mucinous colon cancer (NMCC) tissue was used to develop ex vivo epithelial organoid cultures (colonoids) based on a previously published protocol [7, 40]. Human tissue was collected under an Institutional Review Board (IRB)-approved protocol. The mucosa was stripped of the underlying muscle layer and tumor tissue fragments were washed, followed by incubation in chelation solution supplemented with ethylene diamine tetraacetic acid (EDTA, 2 mM final concentration). Basal culture medium (advanced DMEM/F12 supplemented with penicillin/streptomycin, 10 mM HEPES, and Glutamax) was added, and the crypts were washed twice with basal culture medium and suspended in Matrigel matrix (MM; Corning, Tewksbury, MA). The MM was polymerized by incubation at 37 °C in a 5% CO_2_ incubator for 30 min and then overlaid with human intestinal stem cell medium.

### Tumor explant culture

Tissue from fresh primary MCC and NMCC was obtained during surgery. The explant culture system was used according to a previously described method [7, 41]. Using a 4-mm biopsy puncher, cubes of tumor tissue were prepared and placed in antibiotic gentamicin containing DMEM and 10% FBS (typically three cubes/well in 24-well plates). The tumor explants were cultured at 37 °C in a humidified atmosphere containing 5% CO_2._

### JC-1 staining for mitochondrial membrane potential

The JC-1 Mitochondrial Membrane Potential Assay kit, (Cayman Chemical, Ann Arbor, MI) was used to monitor mitochondrial transmembrane potential (ΔΨm). Cells were stained with 100 μL/mL JC-1 staining solution in culture medium in a 24-well plate and incubate at 37 °C for 15 min. In the undamaged mitochondria, the aggregated dye induces red fluorescence, whereas in apoptotic cells with altered ΔΨm, the dye remains as monomers in the cytoplasm with diffuse green fluorescence.

### MUC2 ELISA

Conditioned medium from LS174T cells/ Cos-7 cells expressing MUC2 N-terminal (50 μL/well) was coated onto a Corning 96-well EIA/RIA plate by incubating overnight at room temperature in 0.1 M carbonate buffer pH 9.6. Plates were blocked for 1 h at room temperature with 2% bovine serum albumin (BSA) in PBS and incubated overnight with MUC2 antibody in PBS containing 0.05% Tween-20. Bound MUC2 antibody was detected using anti-mouse HRP-conjugated and 2,2′-azino-bis (3-ethylbenzothiazoline-6-sulphonic acid (ABTS) substrate (Sigma-Aldrich St. Louis, MO).

### Fatty acid synthase assay

In brief, cells were collected in assay buffer (100 mM potassium phosphate buffer, 1 mM EDTA, 1 mM PMSF and 1 mM dithiolthreitol, pH 7.0), and sonicated on ice and centrifuged at 12,000 rpm for 30 min at 4 °C, the supernatant was collected for the reaction assay. Fifty microliters of supernatant was added to the reaction mix contained 25 mM KH2PO4-K2HPO4 buffer, 0.25 mM EDTA, 0.25 mM dithiothreitol, 30 μM Ac-CoA, 100 μM Mal-CoA, 350 μM NADPH (pH 7.0) in a total volume of 200 μl. Protein content in the supernatant was determined using a bicinchoninic acid (BCA) assay (Pierce) and results were expressed as the specific activity of FAS (U/mg).

### In vitro acyl-biotin exchange (ABE) assay

The acyl-biotin exchange (ABE) assay used was adapted from Wan and colleagues with modifications [42]. COS-7 Cells were lysed in lysis buffer (LB, pH 7.4) containing 50 mM Tris-HCl pH 7.4, 150 mM NaCl, 1% NP-40, 1 mM EDTA, and protease inhibitors. For this procedure, all centrifugation steps were carried out at 850×*g* for 5 min. MUC2 was first immunoprecipitated from 500 μg protein using 6 μg anti-MUC2 antibody, and the MUC2 and anti-MUC2 antibody complexes were bound to the exosome immunoprecipitation reagent (Protein G, #10612D, Fisher scientific). Then, 50 mM of N-ethylmaleimide (NEM, E3876, Sigma-Aldrich) in LB, pH 7.4, was added to the immunoprecipitated MUC2 and incubated for 3 h at 4 °C with gentle rotation to block free thiols of cysteine residues. After three washes with LB, pH 7.4, MUC2 was treated with and without (mock as control) 1 M hydroxylamine (HAM, #379921, Sigma-Aldrich) in LB, pH 7.4 for 2 h at room temperature with gentle rotation. MUC2 was then rinsed three times with LB, pH 6.2 followed by treatment with 5 μmol BMCC-Biotin (#21900, Thermo Fischer Scientific) in LB (pH 6.2) overnight at 4 °C with gentle rotation. This was followed by three rinses with LB (pH 7.4) to remove excess biotin, and MUC2 was then eluted with reducing sample buffer. Samples were analyzed using SDS-PAGE.

### Murine xenograft model

Nude mice (Taconic Biosciences) were intraperitoneally injected with 5 × 10^5^ LS174T cells which was stably transduced via lentiviral transfection of the pGL4 Luciferase Reporter Vector (Promega) and selected with puromycin. The luciferase signal was monitored by injecting the luciferase substrate luciferin (150 mg/kg, i.p.; GoldBio) 5 min after anesthesia with 2% isoflurane prior to imaging on an IVIS200 system (PerkinElmer). Bioluminescence signal was quantified using the LivingImage software (PerkinElmer). For LS174T xenografts, mice with similar levels of bioluminescence were divided into four groups (6 animals per group) including Control, Celecoxib (20 mg/kg, IP) alone, Orlistat (10 mg/kg, IP) alone, or Celecoxib + Orlistat combination. Each group of mice was treated every other day. The tumor load was checked from week 1 to week 5 via the IVIS bioluminescent Imaging System. All animal experiments were carried out at the University of Pittsburgh (Pittsburgh, PA, USA) in accordance with the Guide for the Care and Use of Laboratory Animals.

### Statistical analysis

GraphPad Prism 5 software (GraphPad Software) was used for statistical analysis. Two-group comparisons were performed using the Student *t*-test. Comparisons among more than two groups were assessed using an analysis of variance (ANOVA) with post hoc testing.

## Data Availability

The data that support the findings of this study are available on request from the corresponding author.

## Abbreviations

ATF4: Activating transcription factor 4
ATF6: Activating transcription factor 6
CHOP: CCAAT-enhancer-binding protein homologous protein
CIMP: CpG island methylator phenotype
COX-2: Cyclooxygenase-2
ERAD, DMEM: Dulbecco’s modified eagle’s medium
ER: Associated protein degradation
ERS: Endoplasmic reticulum stress
FAP: Familial adenomatous polyposis
FASN: Fatty acid synthase
GNAS: Guanine nucleotide binding protein G_s_alpha subunit
GRP78(BiP): Glucose-regulated protein, 78kD/binding immunoglobulin protein)
IP: Intraperitoneal
IRE1: Inositol-requiring enzyme 1
MUC2: Mucin 2
PARP1: Poly ADP-ribose polymerase 1
PDK1: Pyruvate dehydrogenase kinase 1
PERK: Protein kinase activated by double-stranded RNA-like ER kinase
PDX: Patient-derived xenograft
PUMA: p53-upregulated modulator of apoptosis
RAS: Rat sarcoma
SERCA: Sarcomplasmic endoplasmic reticulum calcium ATPase
TCF4: Transcription factor 4)
UPR: Upregulated protein response
